# Trace Minerals and Livestock: Not Too Much Not Too Little

**DOI:** 10.5402/2012/704825

**Published:** 2012-12-04

**Authors:** Marta López-Alonso

**Affiliations:** Department of Animal Pathology, Veterinary Faculty, 27002 Lugo, Spain

## Abstract

The new approaches of the animal production systems make managing the mineral nutrition a challenge. Versus the excessive, trace mineral supply in intensively managed livestock, well above the physiological requirements, is the no trace mineral supplementation of organic systems, which become highly dependent on trace minerals in the soil. Nowadays, in addition to the animal health perspective, trace mineral nutrition must be environment friendly and allow getting mineral-enriched animal products. We are in a new scenario, where a balance between animal trace mineral needs and limits is needed. This papers focuses on different aspects that will help us to enter a critical dialogue in relation to animal-human-environment.

## 1. Introduction

It is well known that trace minerals (Co, Cu, Fe, I, Mn, Mo, Se, and Zn, among others) are required for the normal functioning of basically all biochemical processes in the body. They are part of numerous enzymes and coordinate a great number of biological processes, and consequently they are essential to maintain animal health and productivity. Optimal nutrition, with adequate trace mineral levels, guarantees proper functions of the organism, among which the most important are structural, physiological, catalytic, and regulatory [1]. 

Trace minerals must be provided to livestock in optimal concentrations and according to requirements that change during the rapid growth and development of the animal and the production cycle. It is rather difficult to justify the term “requirements” for trace minerals in the same way as it is for energy, protein, or amino acids. Requirements for minerals are hard to establish and most estimates are based on the minimum level required to overcome a deficiency symptom and not necessarily to promote productivity [2]. Many authorities—in example the INRA en France, ACR in the United Kingdom, or FEDNA in Spain—have recommended mineral requirements to ensure that the production of native livestock is not impaired by dietary mineral imbalances; however, agreement is rare. The recommendations for livestock in the USA are perhaps more up to date [3–6]. Mineral recommendations should include a safety margin to take account of the presence of antagonists [7–9]. For example, in ruminants Cu uptake is inhibited particularly by Mo but also by S and to a lesser extent by Fe [1] and high levels of Ca in the feed inhibit the uptake of Zn. It is also established that higher levels of Cu are required in the presence of high levels of Zn and that animals under stress require higher levels of Cu and Zn. Moreover, when determining mineral recommendations, consideration must be given to the quantity and type of raw ingredients and their inherent mineral content, the processing of the diet, the storage and environmental conditions, and the inclusion and content of other minerals [2].

Despite the fact that the role of trace minerals in animal and human health is well established, they are the great forgotten nutrients in animal diets. Their physiological role is often underestimated and their presence in the feed in adequate quantities is taken for granted. However, they are necessary to maintain body function, to optimise growth and reproduction and to stimulate immune response and therefore determine the health status [1]. Indeed, it is difficult to realize the impact of insufficient trace minerals, as symptoms of deficiency or mineral unbalances may not always be evident. However, a slight deficiency of trace minerals can cause a considerable reduction in performance and production. Many trace minerals have very specific but often multiple roles. In example Se, it has been known for a considerable time to be necessary for growth and fertility in animals and for the prevention of a variety of diseases; even though more recently it has been established to form an integral part of a number of enzymes (selenoproteins) most of which function as antioxidants in the cellular cytoplasm in a range of situations [10]. As well as individual trace minerals having several functions, several trace minerals may be involved in a single function. For example, Se, Zn, and Cu are all involved in immune function [11]; this makes that rather than considering the role of each trace element it is more helpful to consider the role of trace minerals as a whole in some of the more common problems encountered in livestock.

Despite being as important as they are in animal nutrition, why are they not given enough importance in practice nutrition?—a look at the literature of trace minerals in the veterinary science could help to understand it. Most of the work on trace mineral metabolism in domestic animals was carried out until the 70–80s. At that time most of the actual knowledge on mineral metabolism in livestock was established and it was brilliantly compiled by Eric John Underwood in the book *The Mineral Nutrition of Livestock,* first edited in 1966 [12] and several times reviewed together with Neville Suttle [13]. From a practical point of view, many areas where mineral deficiencies (in some cases also toxicities) were a problem in animals around the world were identified and treated by using dietary supplementation; by that time most of animal production was conducted in traditional systems, quite linked to soils and local feed, and for this reason mineral imbalances were quite usual and important.

In the last decades there has been an enormous growth in livestock production, driven by an increasing demand for animal-source foods among large segments of the world's population, developing countries accounting for the main share of this increase [14]. Globally, livestock production has responded to the increasing demand primarily through a shift from extensive, small-scale, subsistence, mixed crop, and livestock production systems towards more intensive, large-scale, geographically concentrated, commercially oriented, specialized production units. Monogastric species (pigs and poultry) in particular, by virtue of their high feed conversion ratios and short generation intervals, are well suited to rapid intensification of production. It is estimated that more than half of global pork production and 70 percent of poultry meat is now produced in intensive systems [15]. To maintain a high standard of production, animal nutrition becomes very specialized and is based nearly exclusively on concentrate feed with feedstuffs from the international market. Nutrition was focused on main dietary components (energy and protein) and mineral supplements were routinely incorporated into concentrate feed ensuring that animals receive the required intake. This was possible because concentrate rations can be formulated with large “safety margins” so that nutrient intakes largely exceed requirements without important risk for the animal health [16] and assuming that timely supplementation of complete trace minerals is an inexpensive insurance and is well worth the cost [17]. These large safety margins and the low cost even allowed to use some minerals as growth promoters: the classical example being Cu and Zn supplementation in pigs and poultry [18, 19]. 

Nowadays, the routinely and quite standardised mineral supplementation in concentrate feed—which represents the mainly or even the only feed for intensive systems, but also a complementary feed for many traditional or conventional farms—makes that clinical episodes of mineral deficiencies are infrequent and, consequently the relevance of trace minerals in research and teaching programmes is scarce. 

In recent years, however, perhaps in response to concerns over ethical, health, and environmental issues related to intensive farming methods and, in some cases, associated health scares, we face in a new reality with the development of organic and other sustainable production systems where mineral supplements are not allowed, or at least restricted. Sustainable agriculture can be viewed as ecosystem management of complex interactions among soil, water, plants, animals, climate, and people. The goal is to integrate all these factors into a production system that is appropriate for the environment, the people, and the economic conditions where the farm is located. Farms become and stay environmentally sustainable by imitating natural systems—creating a farm landscape that mimics as closely as possible the complexity of healthy ecosystems. 

Finally, the life-sustaining properties of trace minerals in foods consumed by humans and the associated possibilities for enhancing both functional and market value by changing methods of food production [20, 21] have opened a new area of research on animal nutrition. Lucrative markets for trace element supplements for humans raise concerns that modern diets are inadequate and some claimed that benefits of these supplements supposedly improve fertility, vitality, and resistance to infections [1]. In example, Se is an essential constituent of a number of enzymes, some of which have antioxidant functions. Deficiency of the element in animals makes them susceptible to injury by certain types of oxidative stress. Major benefits of selenium have been found to improve the immune system, protect against cancer, and increase the HDL cholesterol to LDL cholesterol for a healthy heart.

We are then in a new scenario, where a balance between needs and limits is needed. We must guarantee animal health and optimize animal productions together with a respect to the environment and to get animal products with an adequate trace mineral content. This new view of animal trace mineral nutrition could be analyzed under two opposite scenarios: the excess of the intensive systems versus the defect of organic and other sustainable systems.

## 2. The Excess of the Intensive Systems

The livestock sector has a primary and growing role in agriculture economy. Driven by growing populations and incomes, the increase in demand for animal products will be stronger than for most food items: global production of meat is projected to more than double from 229 million tonnes in 1999–2001 to 465 in 2050, and that of milk to increase from 580 to 1043 million tonnes [22]. In this context, intensive production systems have been designed to achieve a very high productivity at a relatively low cost. 

In intensive systems providing adequate amounts of trace minerals to meet animal requirements is critical to maximize health and productivity. There are hundreds of studies in the scientific literature analyzing the effect of different chemical compounds, doses, and protocols on different health and productive parameters in all livestock species and to make a comprehensive review of them goes out of the scope of this paper. From a general nutrition perspective, it is well assumed that including mineral premixes in the concentrate feeds has great benefits because the ratio cost benefit is very low and, even not proved to be necessary because trace mineral concentrations in the feedstuffs are enough, the risk of toxicity is negligible. 

However, this growth of the intensive livestock sector stresses many ecosystems and contributes to global environmental problems. This is because a significant proportion of minerals that are given to livestock are excreted into urine and faeces, and this proportion increases as the margin of mineral supply over mineral requirement increases [1]. Excessive mineral supplies pollute the environment and the chosen or default policy of mineral nutrition on a given farm leaves behind a “mineral footprint” [1]. So, the future of the livestock-environment interface will be shaped by how to resolve the balance of two competing demands: for animal food products on the one hand and for the environmental services on the other [23]. In the same line, high rates of livestock mineral supplementation pursuing enriched-mineral foods, in addition to pose a risk for the environment, could become a risk for consumers because they can lead to a parallel increase of toxic metal residues [24].

### 2.1. Excess of Mineral Supplementation on Animal Health

As previously indicated, mineral supplementation given to livestock reared under intensive production is considered relatively safe because the high degree of dietary standardization and the relatively high security margins for most trace minerals and in most domestic species. For this reason episodes of trace mineral intoxication associated to mineral supplementation are relatively rare; at least a change in the mineral bioavailability or an error in the mineral supplement formulation occurs [16].

In spite of this, especial attention should be paid to particular situations, and possibly one of the best examples is hepatic chronic accumulation in ruminants. This is because of the high difficulty to establish traces mineral requirements in these species depending on other dietary components and the low capacity of these animal species for biliary excretion. In addition, the high heavy metal content of mineral supplements compared to other feedstuffs and their interactions with some essential minerals (especially for Cd, Cu, and Zn) can lead to toxic effects on animals [7, 8, 25].

#### 2.1.1. Hepatic Cu Accumulation in Cattle and Other Ruminants

It is well known that Cu is essential for life processes, as a cofactor for many vital cuproenzymes, but is extremely toxic in excess [26]; because of its dual role, all living organisms have developed highly specialized homeostatic mechanisms to recruit, deliver, and eliminate Cu and to neutralize its toxic effects [27].

Within the domestic animals, there are marked variations in their tolerance to the increased levels of dietary Cu [28]. Whereas pigs are highly tolerant to dietary Cu and it can be supplemented at concentrations as high as 250 mg/kg DM as growth promoters (well above the physiological needs: 3-4 mg/kg DM; NRC; 1998), sheep are the most susceptible species to chronic Cu toxicity and supplementation is restricted to 15 mg/kg DM [29]. It has been demonstrated that sheep, as opposed to pigs, have a limited capacity to accumulate Cu bound to metallothionein in their livers [28] and a very limited capacity to increase biliary Cu excretion in response to the increased Cu intake [30]. Hepatic subcellular distribution studies have demonstrated that the lysosomes Cu storage capacity saturates at lower total hepatic Cu loads [31], leading to chronic Cu concentrations in the nucleus and cytosol at lower exposures that are toxic for other animal species. It is also well known that there are strong genetic differences in susceptibility to Cu accumulation and certain breeds of sheep have been classified as tolerant or resistant to Cu toxicity [1] and as a result it has been possible to improve the resistance of sheep to Cu deficiency and excess by appreciating cross-breeding and selection programmes [32, 33] depending on Cu availability on the diet.

Within the other ruminant species, cattle were traditionally thought to be relatively tolerant to Cu accumulation and reports of Cu poisoning were, until recently, somewhat rare. In fact, Cu deficiency in cattle is a rather common disorder worldwide and cattle diets are regularly supplemented with high Cu concentrations (up to 35 mg/kg DM, the maximum level of Cu supplementation for cattle established by the European Union; [29]) is well above general physiological requirements (10 mg/kg DM; [3]). The relatively wide range for Cu supplementation is because in cattle, and in ruminants in general, Cu nutritional requirements do not depend exclusively on dietary Cu concentrations, but are highly dependent on the Cu availability (Cu from cereals in concentrate feeds is more available than from forage, [4]) and the presence of other dietary elements that can influence Cu absorption and metabolism, mainly Mo, S, Fe, and Zn [1, 4, 34]. In fact, elevated Cu supplementation has, in some cases, been justified in view of the interference of Cu with its antagonists [34]. Even more, some particular conditions should be considered when establishing Cu dietary requirements; for example, in calves the very high Cu absorption efficiency forms milk or in weaned heifers the administration of coccidiostat with Cu supplementation, ignoring the fact that the medication would defaunate the rumen and greatly increase Cu absorbability [1].

It is well documented that chronic Cu poisoning in ruminants is a two-stage process. During the first stage (prehaemolytic phase) Cu accumulates in the liver without clinical signs of disease, and only when the liver storage capacity is overloaded (usually following a stressful event of some sort), an important hepatic damage occurs, leading to the release of high amounts of Cu into the blood stream causing a rupture of erythrocytes (haemolytic phase) [28]. The clinically silent phase of the disease makes diagnosis difficult until the animal suffers the haemolytic crisis. In fact a recent study noted that in herds with episodes of clinical Cu toxicity, the clinical cases represent only a small proportion of the actual cases of Cu poisoning [35]. Identification of animals in this silent prehaemolitic stage of Cu accumulation can be considered very important to avoid not only economic losses due to subsequent severe disease or death, but also to avoid subclinical disease [36].

In recent years an increase in the number of episodes of Cu toxicity has been reported in cattle reared under intensive systems [35, 37–39], even at liver concentrations well below those regarded as toxic in the bibliography [40, 41]. Although in some cases cattle toxicity is associated with an excessive Cu intake in the ration, as well as with changes in the type and bioavailability of dietary Cu supplements [35, 42, 43], in others there is no evidence that Cu supplementation is exceeding the quantities considered safe for this animal species, which highlights the need to redefine Cu needs of cattle reared on intensive conditions [44].

It has also been reported that dietary supplements leading to Cu accumulation in the liver at concentrations only slightly above normal (of around 125 mg/kg wet weight) showed negative effects on animal performance in terms of reduced feed intake and average daily gain [45]. Very recently, results found by our research group have demonstrated that under the conditions of cattle raised in intensive systems in NW Spain, which are similar to those in many European countries, routinely Cu supplementation (15 mg/kg DM) leads to a hepatic Cu accumulation in the liver exceeding the adequate range in most animals, half of them showing hepatic Cu concentrations associated with toxicity [44]; Cu supplementation did not suppose a significant change in the Cu storage capacity and the antioxidant defensive system evaluated by metallothionein and superoxide dismutase expression, but with a significant and important increase of oxidative damage [46]. Liver Cu concentrations that seemingly could be associated with subclinical chronic Cu toxicity, as described in the above-mentioned experiments in cattle, have been described in many countries where Cu supplements are given well above requirements [47, 48] and could have a real impact on animal performance and consequently animal production.

#### 2.1.2. Traces of Toxic Metals in Mineral Supplements

Mineral supplements generally contain trace residues of toxic metals, especially of Cd and Pb [49, 50]. In fact, mineral supplements are considered one of the feedstuffs with the highest toxic metal concentrations, and the maximum admissible concentrations established by the European Union [51] are higher than most of the other feedstuffs [52]. However, its contribution to the total dietary intake is difficult to evaluate since mineral supplements and premixes may be added at different rates according to manufacturer's instruction. EFSA has evaluated the risk of the main toxic metals present in the animal feed on the animal health and it was considered negligible for mineral supplements, if their content is within the maximum admissible levels established by the European Union [49, 50, 53, 54]. The only exception seems to be Cd, not only because its bioaccumulative properties can accumulate at very high concentrations in the liver, but especially in the kidney of adult animals causing adverse effects including renal function impairment, hypertension, disturbance of trace mineral metabolism and acute degenerative damage in the intestinal villi. Minimum toxic levels or maximum safe dietary concentrations cannot be estimated with any precision, since Cd deposition is significantly influenced by dietary interactions with Cu, Zn, Fe, and Ca; thus, in some cases, concentrations of Cd as low as 1 mg/kg in the diet did in fact induce an adverse effect in animals [49].

In addition, a significant increase of Cd accumulation in tissues can be observed when high dietary supplements of trace minerals and Zn are used in the ration [49]. This is due to that these metals have similar chemical properties and are able to induce and compete for the binding sites of metallothioneins. This is especially relevant for pigs, where trace minerals and Zn are used at very high concentrations as growth promoters and have a very high capacity to induce metallothioneins [55]; for example Cd residues in the liver and kidney were double in animals receiving 200 mg trace minerals/kg DM for three months compared to the controls [56]. Recent studies in our laboratory indicate that hepatic and renal Cd accumulation was significantly higher in pigs from intensive systems (Cu and Zn supplemented) compared with extensively grown animals (no supplemented) which was related to the metallothionein induction by the former trace mineral [57, 58].

### 2.2. Mineral Supplementation and Environmental Pollution

In recent years, there has been an increase in the public concern about environmental damage instigated by intensive animal feeding operations [23]. The European Community and other authorities like FAO have set maximum permitted levels for mineral concentrations in foodstuffs in order to protect livestock, the consumer, and/or environment [59]. These follow similar precautionary principles to those adopted by producers. For essential trace minerals, the authorities set maximum supplementation levels at concentrations several times greater than minimum requirements [16]. Manufacturers are clearly drawn by these maximum limits rather than animal need, raising mineral concentrations in feeds far above the actual needs of livestock. In other words, the main existence of these maximum permitted levels, which are to protect the environment, has the opposite effect in practice and leads to unnecessarily large “mineral footprints” [1].

Probably the most outstanding species of environmental concern are pig, poultry, and cattle and the most important minerals are Cu and Zn. This is because in both animal species, Cu and Zn are widely used at high levels as growth promoters in the industry. In pigs, dietary concentrations of 150–250 mg copper sulphate/kg and 2500–3000 mg Zn sulphate/kg (more than 25 times the minimum requirements) have been shown to stimulate growth without exposing animals to any risk of poisoning [60]. Pigs excrete approximately 80–95% of Cu and Zn dietary supplements [60] producing metal-enriched manures [61]. Pig slurries typically contain high levels of Cu (ca. 360 mg Cu/kg DM [62] and when spread over the agricultural soils, they lead to increased soil Cu concentrations [18, 63], toxic effects in plants and microorganisms [64] and other livestock species, as ruminants that are very sensitive to chronic hepatic Cu accumulation [65]. In a study of Cu and Zn in the livers of cattle in NW Spain, a positive correlation was found between liver Cu and the density of pig-rearing units [65], and cattle grazing pastures spread with pig slurry have hepatic Cu concentrations above the normally accepted “safe” values [8, 36, 66]. Zn levels are also very high in pig slurries (ca. 500 mg Zn/kg DM; [62]), However, this metal does not appear to pose a risk for ruminants reared on pastures amended with pig slurry due to this element being closely regulated by homeostatic mechanisms. In fact, in the above-mentioned survey in Spain [65] no evidence of Zn accumulation in cattle was ever found.

Great emphasis has been put on strategies for lowering the excretion of Cu and Zn [67]. In Europe, the common practice of adding 250 mg/kg of trace minerals DM to all pigs' rations has been changed, imposing maximum permitted levels of 170 and 25 mg/kg DM in the growing and fattening stages, respectively [29]. A similar problem arises if dietary Zn is used at high levels to reduce the incidence of diarrhea in early weaned pigs. In fact, there are new strategies to reduce this excretion, as, for example, to assess the requirements of Zn and Cu in weaned piglets in relation to diet composition and phytase activity [68]. Another approach is to use organic Cu and Zn sources instead of inorganic ones that are supposed to have a lower bioavailability, but results are not clear [67].

A controversial point is the use of chelated minerals to reduce the outputs in the environment; that feeding hyperavailable mineral sources at reduced dietary concentrations can maintain the performance in both nutritional and supranutritional contexts, while reducing the dispersal of mineral in animal wastes [1]. But, there is little or no evidence that this is true; in fact Veum et al. [69] have compared fecal trace minerals excretion by pigs given copper sulfate at growth-stimulatory levels to that of pigs given chelated Cu at lower levels; the predictable result is a lower fecal Cu excretion with chelated Cu, but the results indicate no difference in availability and that similar environmental protection could be achieved by reducing the permitted level of copper sulphate supplementation. Similarly, it has been suggested that the pharmacological benefits of adding 2-3 g/kg Zn DM as zinc oxide on the performance of early weaned, scouring piglets could be attained with a sless environmental impact by feeding less Zn in a chelated form [70]; however, fecal Zn excretion is determined by dietary Zn concentration rather than source.

Excess mineral output into the environment can be reduced by feeding minerals at levels that meet the best estimates of maximum individual requirements, which means it is necessary to make changes in current commercial and legislative practice [1]. It has been demonstrated that the removal of all supplementary trace minerals from a fattening ration for pigs has only minor effects on performance and carcass quality, and not all are negative [71]. 

### 2.3. Trace Mineral Supplementation and Animal Products

Animal products (including meat, offal, as well as milk and eggs) are considered important sources of trace minerals for humans, their concentration being higher compared to other foods [72–74]. From an animal nutrition point of view, there has been the temptation to overprovide minerals in animal diets to get mineral-enriched animal products. However, it is not always possible to enhance the levels of trace minerals; this is because there are important physiological homeostatic mechanisms that control the absorption-tissue accumulation and excretion of minerals by the organism. The reason for this regulation is the fact that essential trace minerals are also toxic to cellular metabolism when present at uncontrolled quantities. The homeostatic control of essential trace element flux through the body is mediated by the down-regulation of absorption and/or stimulation of excretion (urine, faecal excretion from endogenous sources; [75]) and it becomes active once the dietary supply of essential trace minerals turns from deficient to sufficient amounts; hence it has been largely used to determine the requirement of essential trace minerals. Consequently, determination of mineral concentrations in sensitive tissues (e.g., Zn blood plasma or bone concentrations), activities of metalloenzymes (e.g., alkaline phosphatase or superoxide dismutase), and contents in animal products (e.g., Zn concentrations in eggs or milk) reflect an absence or presence of homeostatic regulation and thus indicate reliably the point of dietary supplies matching the metabolic requirement. 

Homeostatic control at the site of intestine absorption is known for the most important trace minerals as Zn, Cu, and Fe [75] even though important interactions and competitions are known when one of these elements is over-supplied in relation to the others. For most trace minerals, animal tissue concentrations are largely independent of intake, being altered only by massive dietary intervention [76], with dietary supplements within normal ranges of nutrition having little impact [77]; in example, Reist et al. [78] found that supplementing cows with Cu at 10-fold, the daily dietary requirement was necessary to increase milk Cu concentration. 

On the contrary, for other essential trace minerals, like Se, I, and Co, their homeostatic control is mediated mainly by urinary excretion. These elements are absorbed from the intestinal tract at high rates without homeostatic control over a wide range of supply from a deficient to a considerable excess. As homeostatic elimination from the body becomes active after absorption, the respective elements intrude into the organism in a manner proportional to intake, which explains why bodily fluids and tissue contents of these elements usually continue to raise at dietary supplies exceeding requirements, being those elements with a higher potential to achieve enriched-mineral animal products. Knowles et al. [79] have recently reviewed the on-farm available methods to successfully improve Se, I, Fe, and Co/vitamin B12 and folate in milk. 

Iodine is possibly the essential trace element that better exemplifies how supplementing animal diets (with an animal health objective) can act over human population areas showing the same deficiency. I is needed for the production of thyroid hormones which are vitally important for a correct metabolism, especially during pregnancy and infancy [80]. Historically I deficiency has been a common endemic problem worldwide, known as goiter, particularly in populations far from the sea with a low fish consumption (the main source of I) [81–83]. The geographic pattern of I deficiency in farm animals appears to follow the same pattern of human I deficiency around the world [84] and goiter generally occurs in farm animals where ever human goiter is endemic [1, 85]. The increase of the I concentration in the milk after the animal supplementation has been cited as the reason for the eradication of endemic goitre in the UK in the 1960s, which has been labelled as an “accidental public health triumph” by Phillips [86]. I transfer from feed into food of animal origin has been largely evaluated [80, 87] and has demonstrated to be proportional to dietary I [87], being very high in milk (30–40%) and eggs (10–20%) compared with beef (<1%) or pork (ca. 0.3%).

Nowadays, a particular interest surrounds the ability of Se at supranutritional levels to reduce susceptibility to carcinogens [88, 89]. The supplementation of livestock rations with Se raises milk Se in dairy cows [90–92], egg Se in hens [93], and muscle Se in lambs [94], selenomethionine (such as Se yeast) being more effective than inorganic Se to increase Se concentrations in animal products. Most of the additional Se in milk [92] and muscle [94] is present as selenomethionine and the partition of Se in the egg shifts from the yolk to the albumen when Se yeast is used instead of selenite [95]. The success of selenomethionine is attributable to its intact absorption and resistance to degradation but availability for glutathione peroxidase synthesis in the supplemented animal is relatively poor, and turnover rate in muscle is slow [94]. 

The commercial interest of Se and other trace minerals for the food industry, in addition to the direct input to the human diet, has been focused on the potential beneficial effects on the meat properties during the commercial process. During the interval between the slaughter of an animal and the cooking, autolytic changes take place that affect the taste, smell, and visual appearance of the product. The process is essentially one of oxidative deterioration and consequences such as change of colour (e.g., accumulation of metmyoglobin) [96], exudation (“watery pork”), and taint [97] can have a negative effect on retailer and consumer valuation of meat products. Manufacturers of mineral supplements with potential antioxidant properties, including Zn chelates and Se-Met, have been quick to search for evidence that feeding them to livestock enhances the sensory value of meat, measuring everything from pH and shear strength to “tastiness” in blindfolded consumers in the hope of positive outcomes similar to those found with vitamin E supplements. However, these trials have been conducted with Se given in combination with vitamin E [96]. Improvements in storage characteristics have generally been small [97]. For example, a supranutritional inorganic Se supplement produces only minor improvements in the oxidative stability of chicken meat [97]. An organic Se supplement that raised the Se level in a pig ration from 0.18 (high) to 0.48 mg kg^−1^ DM (very high) reduced “drip loss” significantly and more effectively than inorganic Se [98], but whether the reduction of 0.8% in drip loss, only one-third of the total loss, justifies “medication” of the pork is questionable.

However, changing composition is only one step to bringing new foods to market, as their commercial realization will require the initiative and collaboration of scientists, veterinarians, primary producers, and processors responding to market demands. Uptake of future biotechnologies to capture more value inside the farm gate will also be required if dairy industries are to remain competitive [79]. In addition, the effective cost of a treatment that improves product composition is reduced if the scheme is concomitantly part of animal health care, because farmers recover some cost as improved animal health or performance. Dairy cattle nutritionists should be cognisant that an emphasis on increasing concentrations of nutrients in products will add a new factor to the definition of dietary intake requirements. Nutrient requirements should be calculated by a quantitative factorial method to allow higher demand of nutrient-enriched tissues to be added to the usual requirements of growth, maintenance, and production [99]. However, dietary or parenteral supplementation should never exceed levels safe for animal health [79].

## 3. The Defect of Sustainable Systems 

Sustainable production systems, mainly represented by not only organic farming but also integrated and low-input systems, are an alternative to the progressive intensification of industrial production. Principles of sustainable systems are based on a vision where animals should be part of an agricultural system that is environmentally sound, animal friendly, and considering the whole system rather than only optimizing its parts. Sustainable systems use ecologically sound management strategies with the potentiality (beneficially) of respect the physiological and behavioural needs of livestock [100, 101].

In sustainable systems the main source of trace minerals for livestock is the soil itself and the waste materials from the farm which are recycled on to the pasture. The available mineral concentrations in the different soil types are dependent on the concentrations in the parent rock and on the chemistry of the soil [102, 103], and as a consequence, forages in some circumstances may be deficient or imbalanced in some trace minerals [104–109]. In rare cases acute deficiency or toxicity may occur and remedial actions may be complex and difficult. On the contrary, subclinical deficiencies or toxicities, which are very difficult to diagnose but associated with low productivity, are very common worldwide [1, 9, 106].

In addition, the concentrations of trace minerals in plants largely depend on plant genotype, soil environment, climate, and stage of maturity [1]. Trace mineral concentrations are generally higher in the surface soil and decrease with soil depth. In spite of the high concentration of most trace minerals in soils, only a small fraction is available to plants [110] and soil pH is one of the most important factors affecting it. Trace mineral concentrations in pasture also varied seasonally [111, 112]. Leguminous species are generally much richer in macroelements than grasses growing in comparable conditions [1]. Trace elements, notably I, Cu, Zn, Co, and Ni, are also generally higher in leguminous than in gramineous species grown in temperate climates, with Cu and Zn higher in mixed than in pure grass swards [113].

In contrast to conventionally managed farms, in organic systems the main (or even the only) source of livestock feed is locally produced and the use of mineral supplements is restricted [114]. Although not forbidden, mineral supplements are not the first option, they should be regarded as a short-term measure, as it is a largely conventional approach and not in keeping with organic philosophy, trying to devise more natural and sustainable soil and pasture strategies to increase the mineral content in the forage [115] if they are to achieve a mineral balanced dietary intake for livestock [116]. In some occasions, after improving farm management trace mineral deficiencies are still present; so a good diagnostic plan is essential to correctly quantify them. At present the main strategy for improving trace element status in livestock is to analyse the forages and possibly other feeds and to seek a derogation from the appropriate Sector Body to feed a mineral supplement to overcome any deficits highlighted by the analyses. 

Supplementation of trace minerals in organic livestock is most commonly made through the purchased concentrate feed, mineral premixes, free access mineral blocks, or rumen boluses (ruminants); there is also some use of trace element injections, particularly if local inhibitory factors (such as soil Mo affecting Cu uptake) are present. In addition, trace mineral supplements should be tailored to the specific farm needs and should involve minerals with a high biodisponibility. In conventional farms inorganic salts, such as sulphates, carbonates, chlorides, and oxides, have been the compounds more commonly used as mineral supplements. These salts are broken down in the digestive tract to form free ions and are then absorbed. However, free ions are very reactive and can form complexes with other dietary molecules, which are difficult to absorb. Large quantities of undigested minerals are then excreted and cause environmental pollution. For this reason, there is a growing interest in organic farming for proteinated or chelated trace minerals. In this form, the trace minerals are chemically bound to a chelating agent or ligand, usually a mixture of amino acids or small peptides. This makes them more bioavailable and bioactive and provides the animal with a metabolic advantage that often results in an improved performance. They can therefore be included at much lower levels without compromising performance, thus minimizing nutrient excretion and environmental impact [21].

The high restriction of livestock mineral supplementation organic farming is very positive from an environmental point of view because the effluent emissions to the farm ecosystem are significantly lower, but can turn negative from a human nutrition perspective since as previously indicated some trace mineral concentrations in animal products are related to animal dietary concentration. Finally, in land-based systems soil ingestion when grazing can significantly contribute to livestock trace mineral exposure, and this deserves a special consideration for some elements (including toxic metals) that can be at high concentrations and/or are very available in the soils.

### 3.1. Implications for Animal Health

As indicated, sustainable farming is highly dependent on mineral concentrations in the underlying soil. If mineral concentrations in the soil are balanced and adequate no important mineral related disturbances would be expected, even though it is possibly that a maximum level of production would be not reached. However, a great number or soils around the world show mineral deficiencies or unbalances in a greater or lesser extend [117] and trace mineral disturbances are considered one of the main farm problems for most of the authors that have reviewed the current situation of animal health in organic farming in Europe in the last years [102, 106, 118–123].

The way to deal with trace mineral needs in organic farming will mainly depend on the own-farm husbandry and management practices. Livestock practices are highly standardized in intensive farming, but are very variable in organic, in most cases, farms being the result of conventional units adapting their husbandry and management practices to the more regulated and sustainable methods required to achieving “organic” status. 

On exclusively free-range grazing systems animals are totally/absolutely dependent of trace minerals on pastures. Animals are infrequently yarded and there are limited opportunities to administer trace element supplements via feed and concentrates; so scientists and farmers have developed efficient strategies to monitor, treat, and prevent these deficiencies. Preferred supplementation methods for sheep and cattle have efficiencies of 6 to 12 months and usually involve long-acting injections, slow release intraruminal boluses, and trace element-amended fertilizers; some additional options are available for dairy cows, including soluble minerals introduced into the water supply and dietary supplements if the herd has access to in-shed feeders or feedpad facilities [124]. If organic farmers are to avoid using drenches or injections and reject topdressing pasture with soluble salts, then their alternatives are applying organic fertilizers such as animal manure and seaweed, modifying the pasture composition to include species rich in trace elements, or administering homoeopathic remedies to animals. New Zealand is possibly the best example of this model: the agricultural conventional sector has a long experience to deal with mineral deficiencies that has been transferred to organic [125]. Organic farming is growing in other free-range grazing countries [126] that could make profit of the New Zealand experience. 

Greater difficulties to deal with trace mineral unbalances can arise, as far we are concerned, when a farm comes from a conventional system where mineral-supplemented complementary feed (mainly as concentrate feed) was routinely given to the animals. Whether trace mineral deficiencies exits in these areas is possibly not well known by conventional practitioners because they were well compensated by the minerals contained in the complementary feed; This is the experience we are experimenting in NW Spain. In a study conducted by our research group in beef calves from different production systems within the same areas only mineral deficiencies were observed in the organic farms that did not receive supplementary feed, these deficiencies were not observed in the conventional farms located in the neighborhood when receiving at least a 10% of the diet as mineral-supplemented concentrate feed [9]. The fact that in most cases these mineral deficiencies are subclinical or have not clear symptoms, and that the clinical veterinaries have no experience to deal with them, makes diagnosis difficult. In addition, mineral deficiencies or unbalances can be exacerbated at times when the natural farm resources are not able to guarantee an adequate level of nutrition [127]. 

There is a dearth of reported studies worldwide comparing the trace element status in conventional and organic systems that allow us to have a precise estimation of the degree of mineral deficiency linked to the management and husbandry organic practices. In New Zealand, it is well known that the majority of sheep, cattle, and deer have a high prevalence for Co, Cu I, and Se deficiency [1], mineral deficiencies being a challenge for organic farming [125]. In Norway, Govasmark et al. [108, 128, 129] evaluated the mineral status in organic dairy cattle farms and found that herbage Se and Zn concentrations were inadequate to meet the dietary Se requirements; consequently, supplementation was recommended; in addition Cu, Mo, and Co were not correctly balanced to meet the needs of ruminants which could cause mineral interactions. Roderick and Hovi [118] in the UK evaluated animal health and welfare for the Ministry of Agriculture and found mineral deficiencies for most livestock species including poultry. Mineral deficiencies, notably trace minerals, Se, and Co showed a regional pattern and were considered as one of the main problems of the farm by the majority of the producers surveyed. Our experience in NW Spain indicates that organic beef [9] and dairy cattle (unpublished data) and extensively grown pigs [57] showed a lower mineral status compared with intensively grown animals; Co, Cu, and Se deficiencies were only observed in beef cattle that did not receive complementary feed [9], but preliminary data indicates that these deficiencies were more prevalent on dairy farms, possibly due to their higher requirements for milk production.

### 3.2. Assessment of Trace Mineral Status

In conventional agriculture trace mineral farm assessment, with the purpose of designing an own-farm mineral supplementation program, is not as frequent as it could be thought, probably because other approaches in regard to mineral supplementation are considered. The first is to assume that the cost of trace mineral mixes does not justify their use; at this point any reduction in animal performance and feed cost savings is accepted. The second is to assume that timely supplementation of complete trace minerals is an inexpensive insurance and is well worth the cost. In organic systems, the situation is completely different: trace mineral deficiencies are very frequent but mineral supplementation is restricted and should not be used at least that demonstrated necessary for the animal health. For this reason, assessment of trace mineral status is considered a priority in the farm guide of good practices [118].

Great care needs to be taken when assessing the trace element status of grazing livestock. In addition to the high variability on the mineral content in forages and other own farm feeds, in grazing systems it is not possible to do a precise and straightforward estimation of the animal mineral intake (as a function of the mineral content in the feed and the amount of feed consumed) as well as the mineral digestibility. Besides, it appears that marginal trace element status often does not result in clearly defined symptoms, which could help to guide mineral unbalance diagnosis. 

Perhaps the best starting point to assess mineral status for organic systems is the forage, both grazed and conserved, because it represents the main source of feed. However, note should be made of the stage of growth, weather conditions, time of year, composition of the sward, and so forth, as these are all likely to affect trace mineral levels. Such analyses should give a good indication as to whether the animals are likely to have adequate, marginal, or deficient levels of a particular trace element [16].

Assessment of the trace element status in the animal is much more difficult. Account needs to be taken of its point in the production cycle, the level of stress imposed on the animal, the choice of analysis, the level of trace element antagonists as well as other trace minerals in the feed, and indeed the nature of any supplementary feeds used. For example blood Cu concentrations are not good indicators of the Cu status as not all the Cu is available to the animal and is not correlated with Cu concentrations in the liver [130] the best indicator of Cu status: cattle with low plasma Cu levels can have adequate liver Cu levels [131]. Cu concentrations are also highly influenced by antagonist (such as S, Mo, Fe, or Zn), infection, trauma, and stage of production and hepatic Cu concentrations largely vary with stage in the production cycle, declining during the preparturient period reaching its lowest at calving and increasing after calving [1, 132].

### 3.3. Good Practices to Improve Livestock Mineral Status

Conventional farming has numerous tools to deal with trace mineral deficiencies and/or imbalances, for example offering the animals a complementary winter feeding, improving pasture production by using fertilizers and pesticides, or having a pharmacological control of parasite infections, measures that directly or indirectly help to maintain or even improve the animal mineral status. On the contrary, on organic systems most of these farming practices are not allowed or are economically unviable, which force to focus on a highly efficient grazing management to get an adequate mineral status. An integral management of the farm should consider aspects related to the soil, pasture, and animal to ensure an adequate mineral nutrition.

#### 3.3.1. Soil Management

Topsoil is the capital reserve of every farm and “feed the soil to feed the plant” is often referred to as one of the principles of organic farming [133]. It is absolutely essential that all nutrients removed in farm-produced products are replaced. Nutrient advice should be formulated on the basis of soil fertility levels, stocking rate, and crop nutrient requirements and nutrient management in organic farming systems should be based on regular soil nutrient analysis and nutrient budgets which are used to plan applications of manures, composts, and permitted fertilisers [134]. The balance between the different nutrients is very important and maintaining it is more difficult in organic farming compared with conventional one because the regulations do not permit the use of manufactured fertilizers for correcting such an imbalance except in extreme situations. A high level of management skill is needed to put an effective system of actions in place that will ensure an adequate supply of trace minerals for livestock. 

Soil organic matter is strongly associated with the transfer of trace minerals from the soil to the plant [135]. From 98 to 99% of Cu, 84 to 99% of Mn, and 75% of Zn are carried on organic complexes within the soil [136]). A low livestock density (1.0 to 1.5 LSU/ha) prevents overgrazing and a reduction of organic matter input to the soil. Excessive liming causes a reduction in soil organic matter; the soil pH should be at 6.5 or slightly below. Adequate moisture supply should be available to pasture herbage but overdrainage in pastureland should be avoided. 

The soil pH affects the availability of trace minerals for uptake by pasture species. Overtime leaching by rainfall causes the soil pH to fall and this affects the balance of trace minerals in the soil. A soil pH of 6.5 is considered to be the optimum for a soil containing trace minerals in well-balanced amounts. At soil pH values below 6.5 the availability of Mo and Se is reduced and the availability of Fe, Mn, Co, Zn, and B is increased; the opposite is true at soil pH values above 6.5 [137]. Soils that are high in Mo and Se should be limed to a pH which does not exceed 6.0 to reduce the risk of induced Cu deficiency or Se toxicity in livestock. The pH of soils which are low in Mn, Co, Zn, and B should not be allowed to exceed 6.0. Overliming of soils low in Co can result in Co deficiency and ill thrift in sheep. A high soil pH can also cause B deficiency, resulting in the poor establishment of clover [138] which is essential for the provision of N in organic systems. Severe Zn deficiency results in poor pasture growth, which is due in part to thepoor utilization of N within the plant when Zn is low [136]. 

Free draining sandy or gravelly soils are low in trace minerals. The low status of these soils is associated with low clay, oxide, and organic matter content rather than with the leaching of trace minerals from the soil profile [139]. The exceptions are Se, I, and B, which are relatively easily leached by rainfall. Application of farmyard manure and inclusion of species containing high concentrations of trace minerals in the pasture can increase the supply of trace minerals to grazing livestock. Various measures can be taken to control moisture loss in dry weather; these include the use of dams, sluices, and subirrigation through the existing drainage canals [140]. On the contrary, poor drainage can increase the concentrations of some trace minerals in soil and in pasture species [141]. Especial attention should be given to high Mo and Fe concentrations, which can induce Cu deficiency in livestock. 

#### 3.3.2. Pasture and Grazing Management

The inclusion of grass and herb species with high concentrations of trace minerals in pasture has been proposed as an efficient tool to increase the intake of trace minerals in livestock [10]. Scientific research into individual herbs species, their mineral concentrations, and their availability is still limited. In particular rough stalked meadow grass and clover have high trace element concentrations and should be included in grass seed mixtures. Chicory, sheep's sorrel, plantain, dandelion and Lotus species (trefoils) have all been investigated to some extent as sources of minerals in livestock diets; these last species because of their high tannin content could be useful in the control of parasite infections as discussed later. A number of seed houses are now offering seed mixes which include a range of grasses, clovers, trefoils, and herbs. While these mixes are generally significantly more expensive and take longer to establish, the indications are that they are much longer lasting. If they also result in better trace element status in the livestock it is likely that the extra cost will be more than offset by a combination of less frequent re-seeding and better animal health. However, before any reliable recommendations can be made we need to understand much more about how the level of each trace element varies in different parts of the plant as well as how it varies with stage of growth through the season. 

A correctly designed and implemented crop rotation is at the heart of organic crop production. It is well known that organic farming without crop rotation is effectively impossible on the long run because of detrimental effects on soil fertility, weeds, and plant health [142]. In addition, rotations and cover crops may significantly contribute to erosion control, another important agricultural problem field. Especially grass-clover mixtures play a crucial role in crop rotations with respect to nutrient management, soil organic matter accumulation and microbial activity, and problem weed management. In addition, the role of oats and certain brassica crops for the reduction and management of weeds and fungal and nematode diseases should not be underestimated. 

Regardless of the species or class of grazing animal, graze management is a key point in the organic systems, and this should emphasize on maximizing dry matter intake from pasture [134]. Efficient grazing practices can make the farm more profitable in several ways: reduce purchased grain costs, reduce mechanical forage harvesting and associated fossil fuel costs, reduce mechanical manure spreading costs, and lower fertilizer costs as manure nutrients are returned to the soil. The higher an animal's requirements are, based on production level, the more important maximizing intake becomes. Thus, lactating dairy cows are the kind and class of livestock that are most sensitive to factors influencing intake. The management of the pastures, as well as the physical attributes of the pasture (too tall, too short, no clover, too much stubble, etc.), is the key to animal production. The components of pasture that we need to be concerned about are plant density, number of tillers/plant, the height of the grass, and species composition. 

#### 3.3.3. Issues Related to Complementary Feed

Ideally, sustainable systems should provide as most as feed as possible from the own farm and land based, reducing at maximum the purchased feed (usually concentrate feed) at moments when local or own-farm feedstuffs are not available (i.e., winter feeding) or to achieve a high level of production (i.e., milk production). Organic legislation is restrictive on this respect: at least 50% of the feed shall come from the farm unit itself and in addition in the case of herbivores, at least 60% of dry matter in daily rations shall consist of roughage, fresh, or dried fodder or silage [114]. 

When used in organic systems, purchased concentrate feed represents an important source of trace minerals for livestock. It is well known that most trace minerals are present at higher concentrations in cereals than in forages, their bioavailability being also higher in the former [1]. If used as complementary feed, the concentrate feed is an excellent tool to improve trace mineral uptake when the requirements of the animals cannot be met by improving husbandry practices. Mineral premixes included in the concentrate should be tailored to the own farm needs and based on chemical analysis of the farm. As far we are concerned, the market supply of organic concentrate feed is still limited in many countries, including Spain; finding competitive organic feedstuffs (good relation nutritional value and prize) is one of the main concerns of farmers nowadays who demand new valuable alternatives. In an example, seaweed supplements in livestock are considered a natural way to improve I status in conventionally grown animals [143–145]. In addition to I, seaweeds are rich in a full range of trace elements, aminoacids, and vitamins [146, 147] and contain bioactive polysaccharides, carotenes, fucoidans, tocopherols, and unsaturated fatty acids with antioxidant, antimicrobial and immunomodulatory activities [148] which makes them very attractive as a microelement feed supplement for organic nutrition [149, 150].

#### 3.3.4. Parasite Control

In contrast to conventional farms, parasite control in organic farms is affected by several of the prescribed changes in management (e.g., access to the outdoors in the summer) and in most countries a ban on preventive medication including the use of antiparasiticides. It is well known that parasites can largely enhance trace mineral deficiencies [1] and on the other hand, some trace minerals, including Co (used to synthesize vitamin B12) and Fe, can play a key role in affecting ruminant susceptibility [151]. 

At present, the nonchemotherapeutic control of parasites is based mainly on grazing management strategies [152]. Preventive strategies, where young previously unexposed stock, are turned out on parasite-free pastures. Evasive strategies aim at avoiding disease producing infections of a contaminated area by moving to a clean area and may be also relevant. An effective control of nematodes can be also achieved by repeated moves of the herd or alternate grazing with other species. In general, high stocking rates are linked with increased parasite loads; lowering the animal density serves two purposes: it reduces the amount of manure in a given area and the residual grazing height of the forage is often much higher, which significantly reduces the probability of parasite infection (80% of parasites live in the first 5 cm of forage aboveground) [151].

Bioactive forages can be characterized as forages containing metabolites that may reduce the establishment of incoming nematodes or reduce existing worm burdens. Initial work has focused on plants with a high content of condensed tannins, like sulla (*Hedysarum coronarium*) and the trefoils (*Lotus spp*.) [153]. Chicory, a plant with low tannin content but high soluble carbohydrate and minerals which may enable the host to tolerate parasites, has been shown to offer animals some protection from internal parasitism [154]. 

Control of nematodes by larvae-trapping fungi, or perhaps in the future by egg-destroying fungi, looks promising for ruminants and certain monogastric animals but delivery systems and practical dosing regimens integrated with grazing management have to be developed [155].

#### 3.3.5. Animal Genetics

A key point when considering the sustainability of a farm system is the livestock ability to adapt to the local conditions. Related to trace minerals, a good example is Cu metabolism in ruminants: it is well known that there is a great genetic variation in their tolerance or resistance to dietary Cu, certain breeds being classified as tolerant or resistant to Cu toxicity [1] and as a result, it has been possible to improve the resistance of sheep to Cu deficiency and excess by appropriate cross-breeding and selection programs [32, 33]. In cattle, it has been reported that Simmental and Charolais may have higher Cu requirements than other breeds such as Aberdeen Angus, and that Jersey cows are more susceptible to Cu toxicosis than Hosltein Friesians [156]. 

A further consequence of the spread of intensive production is a notable loss of animal genetic diversity. Holstein Friesian cattle, for example, have spread to some 164 countries and Large White bread of pig is now present in 139 countries [157]. These breeds, intensively selected by productive characters, have been found not to have adequate morphological or functional characters when moving to free-range grazing systems that are not able to satisfy their higher nutritional requirements [158]. As a consequence, these highly selected animals are not able to express their productive potential and are much more susceptible to develop different metabolic disorders; some of them are very related to trace mineral unbalances: immunity and reproduction are strongly connected with I, Se, and Cu among others [1].

### 3.4. Issues Related to Soil Consumption and Mineral Status

In extensive or land-based systems soil ingestion when grazing can significantly contribute to livestock trace mineral exposure. This is because soil metal concentrations are one or two orders of magnitude higher than in forage and in concentrate feed [159]. Soil ingestion in farm animals occurs mainly during grazing, either as an inadvertent intake due to soil adhered to vegetation or as an active ingestion in response to a lack of essential mineral elements such as Cu, Co, and Mn in the diet [159]. Soil ingestion in ruminants have been investigated to a much greater extent than in other species and can represent up to 18% of organic matter in cattle [160] and even more than 50% in sheep [159]. In horses, soil ingestion has been mostly connected with mass loading of the intestines and digestive disorders, while in pigs, especially piglets, soil ingestion has been associated with Fe supply and can be very high (from 19 to 94%; [161]) particularly when animals are not fitted with nose rings. Soil adhered to vegetation can be very high (25–35% of DM) when grazing animals are present, while on nongrazed plots the soil adhered to vegetation is reported to be none or very low; grazing sheep cause more soil on vegetation than cattle. Soil ingestion will increase with low amounts of forage available, winter season conditions, high stocking rates, root intake, loose soils, and any management that produces pasture conditions with soil contaminating grass.

Despite the potential importance of soil ingestion, little is known about the availability to the absorption of soil-borne metals by livestock. A marked increase of the concentration of several mineral elements like Cu, Mn, Se, and Fe in the diet when adding a certain amount of soil (14%) into the diet of sheep was demonstrated by Healy [162]. Co is included in vitamin B_12_ but very little of Co is taken up by plant; therefore, it is mainly by soil ingestion that sheep and cattle can fulfill their needs of this trace mineral [162–164]. Abrahams and Steigmajer [165] point that even when soil-plant transfers are not so low, ingested soil can occasionally supply greater than 60% of trace minerals (i.e., Cu, and Zn) in grazing sheep. In beef cattle from extensively no mineral-supplemented systems a strong correlation exits between the degree of grazing activity and the Fe status, especially for organic farms [9].

In pigs, the soil contribution to the Fe needs has been well demonstrated. New-born suckling piglets will be deficient in Fe and they will very soon develop anemia if they do not have Fe administered in some way. There is no point in feeding the sow extra Fe as practically nothing will be transferred to the milk. However, piglets display rooting behavior from the day of birth which, under natural situations, will imply soil ingestion and thus an Fe supply from soil [159, 166]. In adult animals, a recent study of our group found significantly higher hepatic Fe concentrations in pigs from sylvopastoral systems compared with intensive [57].

Soil ingestion may be an important pathway for toxic trace minerals animal exposure, both affecting animal health and carrying out to meat, milk, and other animal products. However, little research has been conducted to date on the bioaccessibility of ingested soil-borne contaminant metals and the extent to which these elements are absorbed into the body of grazing stock. Our own research in NW Spain indicates that soil ingestion explains the higher toxic metal As, Hg, and Pb residues found in extensively grown pigs and organic beef cattle [57, 167] even though dietary toxic metal exposure was comparable to that in intensively reared animals. NW Spain is a relatively unpolluted rural area and toxic metal concentrations found in animals from sustainable systems do not pose any significant risk for human health since toxic metal residues were well within the allowed limits if the EU [59], however special attention should be put in contaminated areas. In example, Smith et al. [168] recently evaluated Pb exposure by grazing sheep from a polluted area, considering the relative importance of the soil-plant-animal and soil-animal pathways. With sheep ingesting soil at rates varying according to season from 0.1% to 44% or more of dry matter intake, the soil-animal pathway accounts for the majority of Pb consumption through most of the year, and at moderately and highly contaminated sites significant quantities of relatively soluble soil-Pb can be ingested at rates exceeding safety threshold limits. But whilst metal concentrations in pasture vegetation reflect the high concentrations in the soils, uptake and translocation into the aboveground plant tissues are limited, especially for Pb, for which a variety of soil and/or plant mechanisms are thought to be effectively responsible. The low-relative accumulation of Pb in pasture plants compared to the high degree of soil contamination of vegetation surfaces evident, especially in the winter months, increases the importance of the direct soil-animal pathway in the exposure of grazing sheep to the elevated soil-Pb concentrations. Some measures have been proposed to reduce soil-animal toxic metal exposure in polluted areas including the utilization of an effective rotation of livestock, a reduction of the stocking rate, and/or provision of supplementary feed during the winter months. Geochemical mapping on the occurrence of trace minerals has demonstrated helpful to indicate regional patterns, relating to the composition of bedrock and soil and to contamination from past and present industry [159].

### 3.5. Human Health Concerns

Pasture-based farm systems that provide fresh and conserved forages and occasional concentrate supplements lead to acceptable and sustainable productivity, as well as being a point of difference to concentrate-based production systems [79]. Consumers generally assume these products as more healthy and nutritious, but this is not always true that is, if the farm is located in a trace mineral deficient area it is quite probably that mineral concentrations in animal food were lesser than those in conventional systems. 

Traditionally, the relevance of animal products with a low mineral content from animals grown in deficient areas was not enough considered, possibly because the interest of trace minerals (I, but especially Se) by the food industry and the human health is quite recent. According to Vitti et al. [169], most European countries have mild-to-moderate I deficiency in their human populations. I intervention programs, such as supplements and modified foods, can provide low cost and high benefits to consumers health. While iodised salt is successful in many countries, other staple foods may also need to be fortified to address national risks of intake insufficiency [170]. Dairy products could offer an additional route for human I intake, particularly in infants and children. Se intake is low in many areas as a consequence of the country's geochemical makeup [171] and milk has demonstrated to be an effective delivery vehicle for human Se intake, especially for children, since methods exist to produce high Se milk using parenteral supplements or feed additives [79].

In the last years the low I concentration found in the organic milk in different countries has opened a public debate [172] about the risk of consumption of this milk for some population sectors as children and pregnant women [80]. Recent studies in Denmark [173], Norway [174], UK [175], and north Spain (López-Alonso, unpublished data) have pointed out that I concentration in the organic milk is significantly lower than that in the conventional (ca. 60%). In all these studies I concentration was even significantly lower in the summer which has been related to the low degree of concentrate supplementation during this season. In addition to the general mineral restrictions in organic farming, as previously indicated, the lower I concentration in the organic milk can be explained, at least in part, by differing practices on the organic and conventional farms. Nitrogen-fixing crops, such as clover, are important in organic farming and are used in place of artificial fertilizers [176]. White clovers contain cyanogenic glucosides that are thought to exhibit goitrogenic properties [177] and, as suggested by Rasmussen et al. [173], the greater use of goitrogenic feed could lower milk-I concentration through the inhibition of the sodium-I symporter in the mammary gland of the cow. In addition, although iodophor disinfectants and teat dips are allowed in organic farming, their use as a chemical product, it is possibly more careful in the organic farming.

## 4. Conclusion

This paper opens a discussion on the need to strike a balance between animal health, human health, and environment pollution to avoid that the “friendly” trace minerals become “foes”. Veterinarians play an important role in this development through work in clinical practice and in research. On-farm consultancy should be tailored to the individual farmers needs, and the practitioner should be willing to take up new ideas, and when needed, to enter a critical dialogue in relation to animal-human-environment.
